# Predicting cryptic links in host-parasite networks

**DOI:** 10.1371/journal.pcbi.1005557

**Published:** 2017-05-25

**Authors:** Tad Dallas, Andrew W Park, John M Drake

**Affiliations:** 1 University of Georgia, Odum School of Ecology, Athens, Georgia, United States of America; 2 University of California, Department of Environmental Science and Policy, Davis, California, United States of America; 3 Center for the Ecology of Infectious Diseases, University of Georgia, Athens, Georgia, United States of America; Imperial College London, UNITED KINGDOM

## Abstract

Networks are a way to represent interactions among one (e.g., social networks) or more (e.g., plant-pollinator networks) classes of nodes. The ability to predict likely, but unobserved, interactions has generated a great deal of interest, and is sometimes referred to as the *link prediction problem*. However, most studies of link prediction have focused on social networks, and have assumed a completely censused network. In biological networks, it is unlikely that all interactions are censused, and ignoring incomplete detection of interactions may lead to biased or incorrect conclusions. Previous attempts to predict network interactions have relied on known properties of network structure, making the approach sensitive to observation errors. This is an obvious shortcoming, as networks are dynamic, and sometimes not well sampled, leading to incomplete detection of links. Here, we develop an algorithm to predict missing links based on conditional probability estimation and associated, node-level features. We validate this algorithm on simulated data, and then apply it to a desert small mammal host-parasite network. Our approach achieves high accuracy on simulated and observed data, providing a simple method to accurately predict missing links in networks without relying on prior knowledge about network structure.

## Author summary

The majority of host-parasite associations are poorly understood or not known at all because the number of associations is so vast. Further, interactions may shift seasonally, or as a function of changing host densities. Consequently, host-parasite networks may be poorly characterized since effects of cryptic host-parasite associations on network structure are unknown. To address this, we developed theory and applied it to empirical data to test the ability of a simple algorithm to predict interactions between hosts and parasites. The algorithm uses host and parasite trait data to train predictive probabilistic models of host-parasite interaction. We tested the accuracy of our approach using simulated networks that vary greatly in their properties, demonstrating high accuracy and robustness. We then applied this algorithm to data on a small mammal host-parasite network, estimated model accuracy, identified host and parasite traits important to prediction, and quantified expected changes to structural properties of the network as a result of link relabeling.

## Introduction

Complex interactions between host and parasite species can be described as a network, with host and parasite species as two distinct node types connected by links that represent associations between a given parasite and host species. Understanding the structure [1] and stability [2, 3] of host-parasite networks is important for establishing drivers of host-parasite interactions, parasite specificity, and the consequences of host extinctions on parasite diversity. Recently, authors have applied concepts and tools from community ecology and graph theory to host-parasite interactions [4–7] in an effort to understand how host and parasite communities interact, including investigations into how host community diversity influences disease transmission [8], how parasites interact within infected hosts [9], and how host functional and phylogenetic similarity promote parasite sharing [10, 11]. Additional research has focused on topological measures of host-parasite networks—such as nestedness [12] and modularity [13]—which attempt to quantify the formation of patterns of interactions between host and parasite species. These patterns may influence network stability [2] and resilience [3]. Identifying the factors influencing the formation of these patterns is an important nascent area of research.

There is little consensus about whether various reported topological patterns are common [14–16], which may be a result of the influence of sampling effort and the effect of incomplete detection on measures of topological network structure [17]. Specifically, the detection of patterns in most studies is predicated on having completely sampled the network of host-parasite interactions. That is, all interactions between host and parasite species are assumed to have been documented in the course of the study. However, such exhaustive sampling is rare at best, as logistical constraints often limit detection of all interactions. Moreover, the total number of potential host-parasite interactions increases as a product of the number of host and parasite species, creating a large number of opportunities for a missed detection of a host-parasite interaction. It is unlikely that studies of ecological networks are recording all of the potential interactions between species, as even long term data have been unable to detect a large number (nearly 50% of plant-pollinator interactions) of species interactions [18]. Incomplete sampling compromises inference of network structure and stability, and may undermine studies of parasite specificity and measures of parasite species richness for a given host species.

Despite this complication, there is a body of research aimed at predicting host-parasite interactions. This work is of clear importance to wildlife and human health—as the it is possible to identify potential spillover events [19–21]—and to a general understanding of the traits associated with parasite specialization. To this end, current approaches examine parasite species independent of the network within which they are embedded, using host traits to predict likely interactions. Two such efforts attempted to predict the fish host community parasitized by helminth parasites [22, 23]. However, approaches to date have not explicitly considered how the distribution of host and parasite traits, or the complex interactions at the host-parasite network level could influence predictability of host-parasite interactions. By considering all potential interactions simultaneously, it is possible to find the most probable interactions given the entire network, rooting the problem of predicting likely host-parasite interactions within a body of theory from the study of complex networks [24, 25].

Here, we address this problem by developing and testing a method capable of determining the number of likely unobserved host-parasite interactions, and accurately predicting the most likely, but undetected, host-parasite interactions in the network. This is not a new problem, as computer scientists have struggled with the *link prediction problem* for decades, most notably in studies of social networks [26–28]. We focus on link prediction in bipartite networks, with a specific application to ecological networks. Previous work in link prediction for bipartite networks has required information on traits of both node classes (e.g., host and parasite species), as well as knowledge of network topology (e.g., degree distribution) [29]. Here, we develop a highly accurate link prediction method based on trait matching between host and parasite species. That is, we make no assumption about network topology, but predict bipartite interactions using only trait data on host and parasite species. We examine the performance of our algorithm on simulated data extensively, and then test the algorithm on an ecological host-parasite network of small mammals and their resident parasite communities in a New Mexican desert ecosystem.

## Methods

### A plug-in approach to conditional density estimation

We propose an approach to identifying cryptic associations in host-parasite networks based on numerical estimation of conditional density functions. We represent the connections between hosts and parasites as a sparse bipartite graph (*H*, *P*, *E*) with vertex sets *H* (host species) and *P* (parasite species) and edges *E*, such that an edge connects *H*_*i*_ and *P*_*j*_ if species *j* parasitizes species *i*. If there is an edge between *H*_*i*_ and *P*_*j*_, we write *y*_*i*,*j*_ = 1 whether the edge has been observed or not; otherwise *y*_*i*,*j*_ = 0. Not all edges have been observed and not all possible edges exist. Thus, *E* consists of both observed edges *E*^*o*^ and unobserved edges *E*^*u*^ = *E*∖*E*^*o*^ and is itself a subset of the possible edges $\tilde{E}=H\times P$. Attached to each host and parasite species are vectors of features *h* and *p*, respectively. Thus, edge (*H*_*i*_, *P*_*j*_) has the combined feature set *x*_*i*,*j*_ = (*h*_*i*_, *p*_*j*_).

To identify cryptic links in *E*^*u*^, we seek a ranking of edges according to their probability. The probability that there is an edge between two vertices given its feature set is written *P*(*y* = 1|*x*). From Bayes’ theorem, we have

$$P\left(y=1|x\right)=\frac{f_{1}\left(x\right)P\left(y=1\right)}{f(x)}$$

where *f*_1_ is the conditional probability of feature set *x*_*i*,*j*_ given that *y*_*i*,*j*_ = 1, *P*(*y* = 1) is the *connectance* of the graph, and *f* is the density of all possible combined feature sets. That is, *f*_1_ is the probability density of features when a link exists between host and parasite, and *f* is the density of features for all possible host-parasite combinations. The model assumes that the observation process (probability of detection) is either constant or random with respect to host and parasite features. Extensions of this model could address this assumption through the incorporation of features related to sampling probabilities or the use of model simulations directly incorporating the observation process. Since we seek only a rank ordering, we ignore *P*(*y* = 1) which is simply a normalizing constant, and estimate *q* = *f*_1_/*f*.

Estimating *q* is a *density-ratio estimation* problem [30]. The plug-in approach we propose, which we call *plug-and-play*, is to separately estimate *f*_1_ and *f* from the features of *E*^*o*^ and $\tilde{E}$ and to take the quotient as required for evaluating any given host-parasite pair, i.e., $\hat{q}=\hat{f_{1}}/\hat{f}$. In practice, we use the kernel density estimator npudens in the np package [31] and the “normal-reference” bandwidth. This nonparametric approach to density-ratio estimation was chosen because it generally performs very well, particularly when the feature set contains a combination of binary and continuous features [32].

The estimated probabilities of all edges in $\tilde{E}\backslash E^{o}$ are then evaluated and ordered. That is, the model outputs the probability of each edge $\tilde{E}\backslash E^{o}$, which can then be ranked by the most probable undetected edge in the set of cryptic links *E*^*u*^. The AUC (area under the receiver operating characteristic) statistic can be calculated by comparing the observed labels and the estimated probabilities. If probabilities need to be translated into binary states, we begin with the most likely cryptic link, and re-label unobserved edges in order until a stopping criterion is met.

### Simulated host-parasite networks

Host-parasite networks were simulated as follows. First, we generated a number (typically *n* = 5) trait values for both host and parasite species by drawing random numbers from a beta distribution, with the two shape parameters (*α* and *β*) drawn from a uniform distribution bounded between 0.5 and 1.5. The beta distribution was chosen for its flexibility and generality to many ecological and epidemiological problems [33, 34], as it is bound between 0 and 1, can take a variety of shapes, and is easily extensible (e.g., beta-binomial modeling; [35]). Then, the probability that host *i* interacts with parasite *j* was given as the outer product of host *h* and parasite *p* trait vectors, calculated as the row-wise product of host and parasite trait matrices, where rows correspond to either host or parasite species and columns are traits. This forms a matrix of *h* rows and *p* columns. This matrix (*M*) was scaled to the unit interval by dividing each value by the maximum value observed. Interactions were assigned probabilistically by conducting single binomial trials with probability *M*_*i*,*j*_. This process was performed iteratively until a specified connectance value was reached (*c* = c*).

$$h=[h_{1},h_{2},\ldots h_{i}]$$

$$p=[p_{1},p_{2},\ldots p_{j}]$$

M=h×p

while(*c* < c*)

$$M_{i,j}=\left\{\begin{array}{cc} 1 & \text{if}\;M_{i,j}>U\left(0,1\right)\\ - & \text{if}\;M_{i,j}<U\left(0,1\right) \end{array}\right.$$

### Model validation on simulated data

To determine how well the plug-and-play model performed, we tested the predictive accuracy of the model on simulated data. We trained models on 80% of the simulated data, and predicted on the remaining 20% test set, i.e., a setup that assumes only 80% of host-parasite associations to have been sampled. (This criterion is relaxed in the Supplemental Materials where we show how the fraction of the network used for model training influenced predictive accuracy; S1 Fig). The AUC statistic was uesd as a measure of predictive accuracy, and examined how model performance was influenced by interaction matrix size, the fraction of realized links (i.e., connectance), the number of traits used to predict species interactions, and the inclusion of binary (e.g., thresholded at the mean) and uninformative (e.g., standard normal variates) traits (see Supplemental Materials for more information). Unless otherwise stated, species interaction matrices were created and predicted using five host and parasite traits each, and a connectance (*c*) of 0.2, which reflects observations of empirical host-parasite networks [36].

First, we determined the predictive accuracy of our model on 1000 randomly generated species interaction networks. To examine the influence of interaction matrix size, we varied host and parasite species richness from 10 to 30, and simulated 50 networks for each host and parasite richness combination. The influence of connectance was examined by creating 1000 species interaction networks with 30 host species and 20 parasite species for each value along a gradient of connectance values from 0.05 to 0.35. To examine the influence of host and parasite trait number, we simulated 1000 species interaction networks for each host and parasite trait number combination between 1 and 20 (total of 20,000 networks). The influence of training the model on binary trait data was examined by creating 1000 species interaction networks created using 20 host and parasite traits, and varying the fraction of those 20 traits that were binary from 5% (1 trait was binary) to 100% (all traits were binary). To determine if the inclusion of random, uninformative traits influenced predictive power, we simulated 1000 species interactions networks with 10 host and parasite traits, and included between 1 and 50 random host and parasite traits (50,000 total species interaction networks). Lastly, we tested predictive accuracy when the model was trained only on random traits by creating species interaction matrices (1000 per treatment) and then shuffling trait values.

The plug-and-play model was able to predict links on simulated bipartite networks with high accuracy (S2 Fig). Further, accuracy was not appreciably reduced by matrix size (S3 Fig), incorporation of binary variables (S4 Fig), number of host and parasite traits (S5 Fig), connectance (S6 Fig), or the incorporation of random variables (S7 and S8 Figs). Specifically, we found that more than three host and parasite traits were needed to have a mean AUC value of 0.9, and training on only a single host and parasite trait resulted in moderate predictive accuracy ($\bar{AUC}$ = 0.72).

### Application to empirical data

We applied the plug-and-play algorithm to data on parasites of small mammals sampled as part of the Sevilleta Long-Term Ecological Research project. We aggregated data from 1992 to 1997 from six sites in three nearby habitats into one interaction matrix. Details of animal sampling and processing are reported elsewhere [4, 37]. Hosts with fewer than five captures over the six year sampling effort were excluded from analysis, resulting in a total of 22 small mammal host species and 87 parasite species, including both macroparasites (e.g., helminths) and microparasites (e.g., coccidians).

Host trait data were obtained from Pantheria [38], supplemented with published literature sources (see Supplemental Table A1 of [4] for more information). Host trait data included life history traits (Table 1), and phylogenetic information. Phylogenetic relationships were estimated using the first five axes of a principal coordinates analysis (PCoA) on the phylogenetic distance matrix obtained using the mammal supertree [39] and the ape R package [40]. Together, these first five PCoA vectors captured 95% of the variance in the eigenvalues, suggesting that most of the information in the phylogeny was captured in these five vectors.

**Table 1:** Description and units of variables used to predict host-parasite network structure.

Trait	Units	Definition	Mean	SE
Adult mass	g	Average adult mass	63.34	13.71
Abundance	no.	Host abundance	177.3	54.53
Diet breadth	no.	Diversity of food eaten	4.04	0.31
Gestation length	days	Duration of fetal growth	28.44	0.83
Home range	km^2^	Area of activity	1.03*e*^−2^	3.30*e*^−3^
Host phylogeny	–	PCoA on phylogenetic distance matrix	–	–
Litter size	no.	Average number of offspring per litter	4.53	0.34
Litter interval	months	Duration of time in between litters	8.28	2.22
Longevity	months	Maximum adult age	62.39	7.28
Parasite genus	–	Parasite genus	–	–
Parasite type	–	Arthropod, helminth or protozoan	–	–
Tissue infected	–	Location inside infected host (I or E)	–	–

Host life history traits included host diet breadth, body mass, home range size, maximum age, and species abundance (Table 1). Parasite trait data included three variables representing the life history and transmission modes of parasites; parasite type (arthropod, protozoan, or helminth), parasite genus (genus), and location (intracellular or extracellular). Some host trait data was unavailable, and we imputed the unavailable data using the randomForest R package [41]. This procedure imputes missing data by first replacing missing values with column averages, and then iteratively updating imputed values based on proximity of observations to one another in the random forest model. Variable importance was determined by permuting each predictor variable 500 times, and determining the reduction in model performance as a result for each permutation. Model accuracy (AUC) was determined through 5-fold cross validation. The final model was trained on all available data.

### Network structure changes with addition of missing links

We then determined the number of likely missing links from the host-parasite network, and sequentially added the most likely links as predicted by our trained model. We used the Abundance-based Coverage Estimator (ACE; [42]), commonly used for species richness estimation, to estimate the number of missing links. ACE is a non-parametric species richness estimator typically applied to communities of free-living organisms ([43, 44]) and has previously been demonstrated to perform well for many different coverage levels and survey designs ([45]). We treat links between known hosts and parasites to be equivalent to organisms in the traditional context, which allows us to estimate the likely number of links missing from the network.

At each link addition, we calculated properties of the network to observe how network structure changed with link addition. Some stuctural properties change obviously and deterministically with link addition (e.g., mean degree and connectance), which we ignore. Rather, we focused on stochastic aspects of network structure, including measures previously related to network stability (nestedness; [3, 14]), aggegration of parasite species among host species (togetherness and variance-to-mean ratio; [46]), and measures of interaction clustering or host-parasite co-occurrence (*C*-score; [47]). The resulting changes to network metrics with model-predicted link addition were compared with changes in network metrics if links were added randomly.

*Nestedness*, quantified as the NODF metric [48], measures the tendency of hosts with few parasites to harbor nested subsets of the parasite communities of parasite species-rich hosts, and has previously been related to network structural stability [3]. Nestedness was quantified relative a null model, as aspects of matrix size and fill alter the raw measure. Further, the use of the standard score (*z*-score) allows a quantification of the magnitude of divergence from a null expectation, which is commonly used for significance testing. Thus, this approach allows us to determine changes in the magnitude of nestedness with link addition relative to a null expectation. We used the sequential swap algorithm to randomize matrix interactions [49], and compared the empirical network to 1000 null networks after each link addition.

*Togetherness* measures the tendency of host species to share parasites, with large values suggesting ecological similarity between hosts may be more important than competition in driving community structure, and small values suggesting the opposite ([12, 50]). The *variance-to-mean ratio* is an index of aggregation traditionally used in studies of single species parasite distributions [46, 51], where larger values indicate more skewed or aggregated parasite burdens. Here, we use it to express the skew in parasite species richness for a range of host species.

Originally used to infer interspecific competition, the *C*-score (or checkerboard score; calculated here as the mean pairwise score for all host species) is more generally a measure of non-independence in species interaction patterns, with large values indicating that species occupy different habitats ([47]). These interaction differences could be a result of interspecific competition, dispersal limitation, or differences in host habitat utilization. In terms of host-parasite networks, this would correspond to parasite communities with little overlap in host use, such that parasite communities are clumped across the range of potential host species.

## Results

The algorithm we develop here was able to accurately predict missing links in bipartite networks based solely on host and parasite traits, both in simulated networks (see Methods paragraph “Model validation on simulated data”), and an empirical network of small mammal host-parasite interactions sampled as part of the Sevilleta LTER.

### Sevilleta host-parasite link prediction

**Fig 1:**
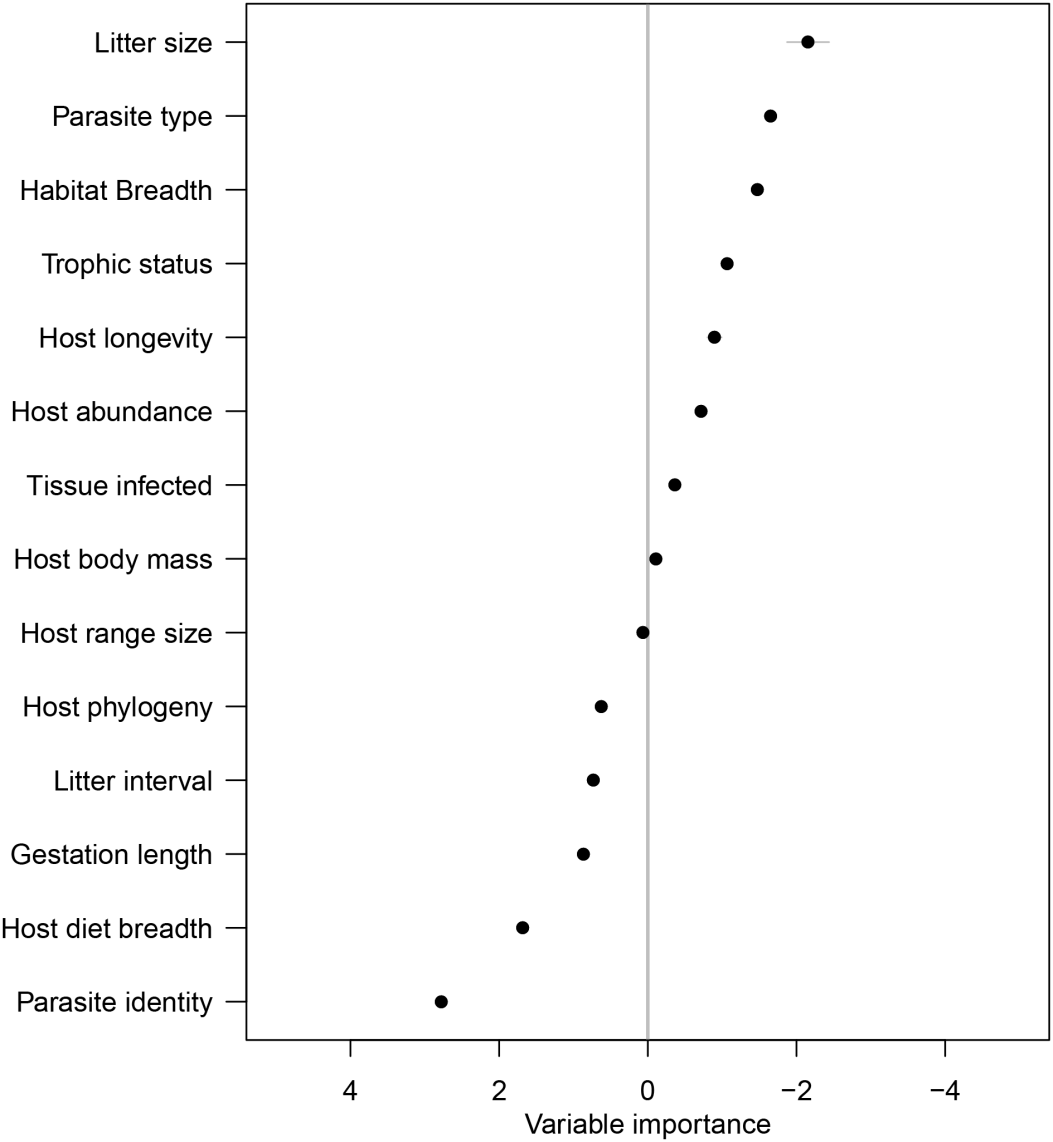
The relative importance of each variable in predicting parasite occurrence in the Sevilleta host-parasite network. Variable importance is measured as the reduction in predictive power by randomizing each variable, and the resulting variable importance scores are *z*-scores. Negative scores correspond to the proportional reduction in model performance as a result of variable randomization. Traits are ordered by importance to the predictive model, with the key predictive covariates in the upper left (e.g., litter size).

The plug-and-play algorithm recovered the Sevilleta small mammal-parasite interaction network structure with high accuracy (AUC = 0.82) when trained on all available data, and performed fairly well during 5-fold cross validation, with a mean AUC from 500 training/test data splits of 0.63, and a maximum observed AUC of 0.81. We permuted predictor variables to obtain measures of variable importance, which suggested that host litter size, parasite genus, and host diet breadth were the most important variables to model performance (Fig 1). Meanwhile, some covariates had a negative effect on the model, resulting in improvement in predictive accuracy with randomization. These included coarse, low-variance variables such as habitat breadth and trophic status, as well as potentially important variables such as parasite type (e.g., helminths), and host body mass. Predictive model accuracy is predicated on the network being fully sampled, such that predicted links that are not observed in the empirical network are treated as errors, and reduce accuracy. We predicted that between 110 and 157 links were missing from the empirical network, changing the connectance from 0.12 to between 0.18 and 0.21.

### Network structure changes with link addition

**Fig 2:**
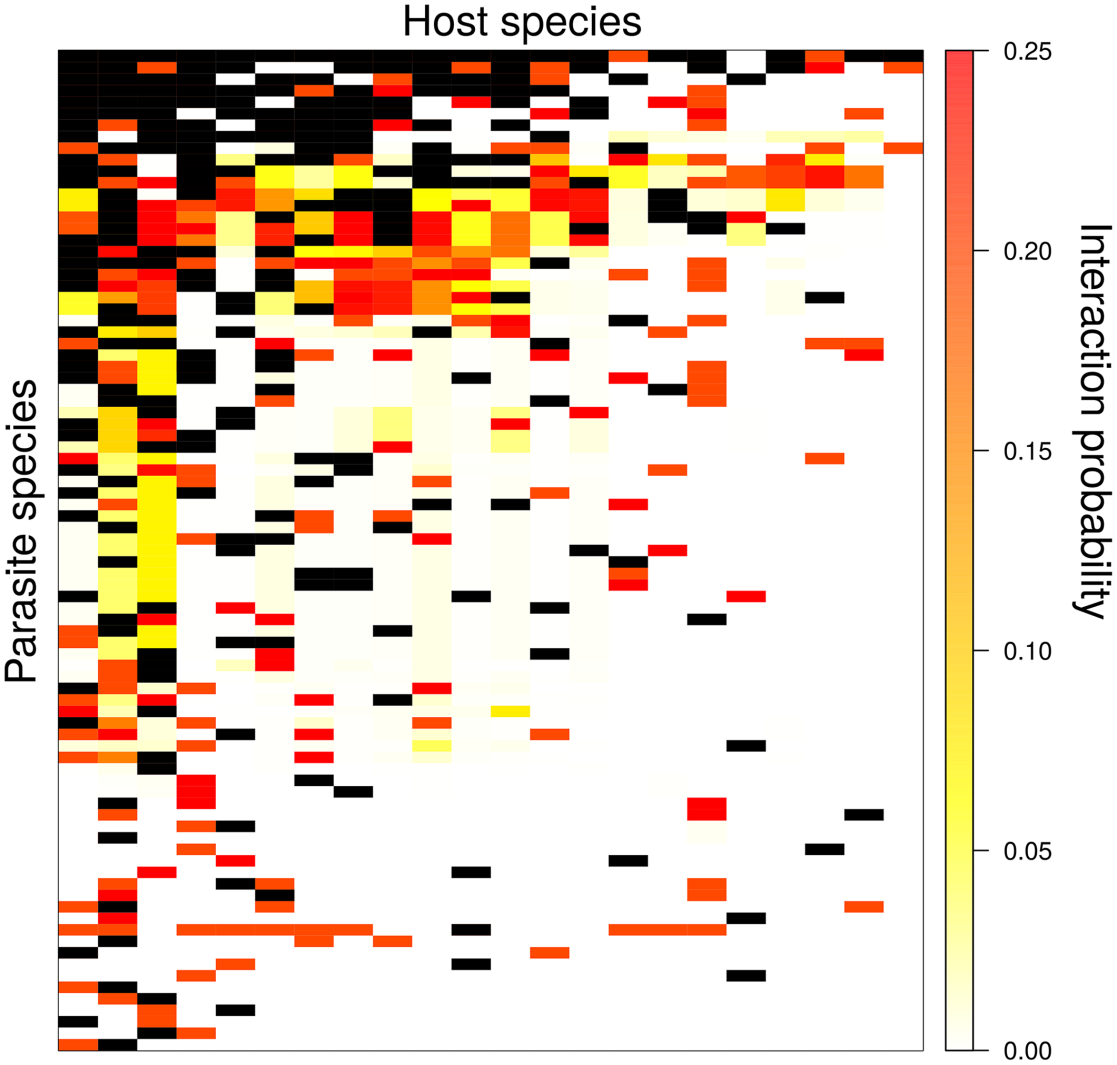
The Sevilleta interaction matrix, where rows correspond to parasite species, and columns to rodent host species. Black boxes indicate an interaction between host and parasite, and color indicates log transformed interaction suitability as determined by the plug-and-play algorithm. Larger suitability values indicate a higher predicted likelihood of an interaction between a host (column) and parasite (row) species.

**Fig 3:**
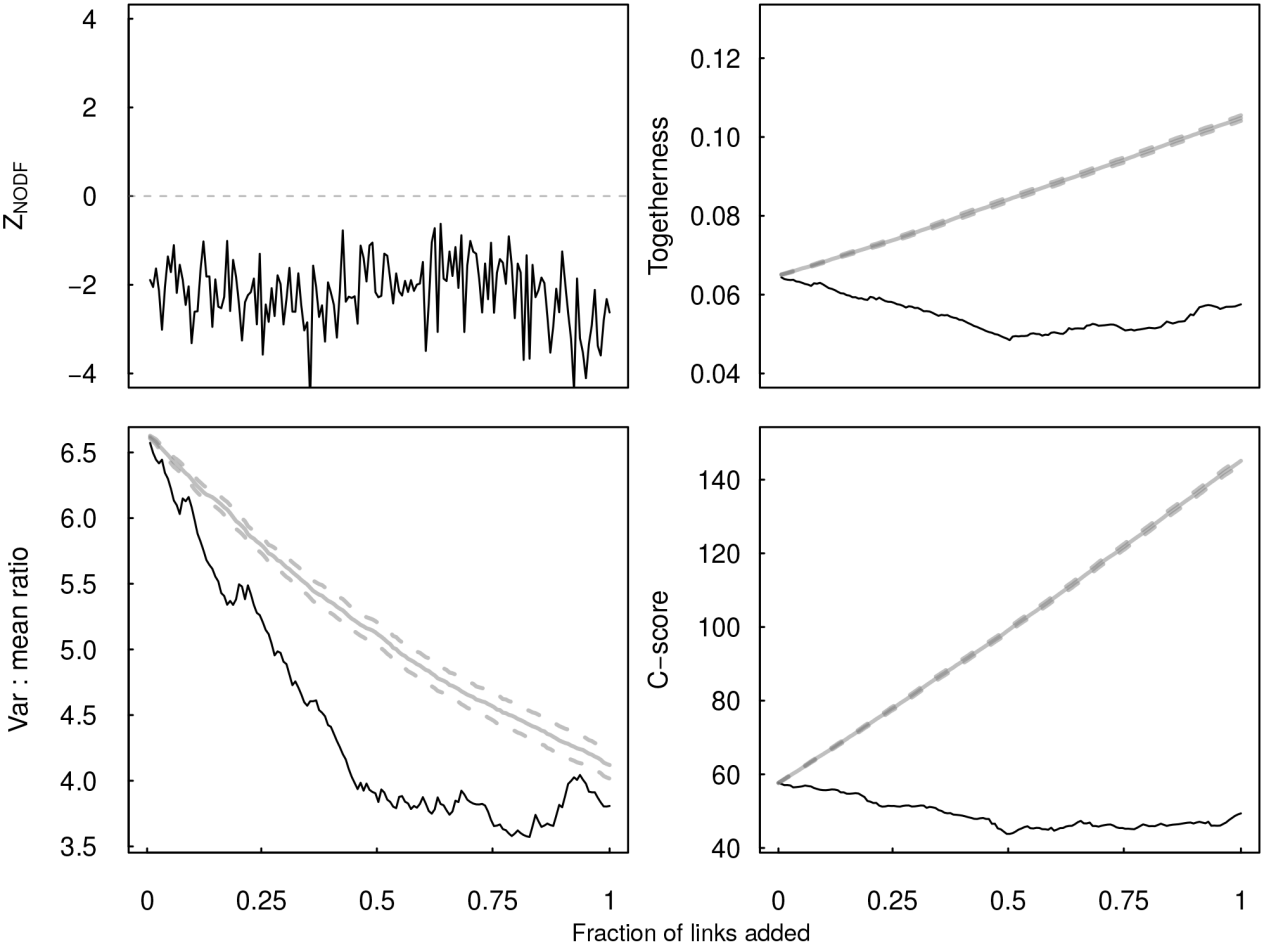
The sequential addition of the most likely missing links resulted in changes to several network properties relative to the change expected under random link addition (grey lines and dashed 95% confidence intervals). Specifically, the ability to detect nestedness (a) fluctuated with link addition. Other patterns showed a much stronger directional signal, including reductions in togetherness, variance-to-mean ratio, and *C*-score. Each of these metrics describes patterns of (dis)aggegration in node degree, suggesting that the fundamental organization of the network changes with the addition of potentially missing links.

We then sequentially added the most probable links, based on model-predicted suitability scores (Fig 2), plug-and-play examine how network properties changed. Measures of network structure fluctuated with link additions (Fig 3). Specifically, nestedness, quantified as the *z*-score in NODF values relative to null models, fluctuated from -4.6 to -0.6. Since these *z*-scores can be used for significance testing, this suggests that the addition of missing links can change the ability to detect fundamental network properties. Further, togetherness, variance-to-mean ratio, and *C*-score all declined more strongly with the addition of predicted missing links compared to the addition of random links. Further, togetherness actually increased when link addition was random.

## Discussion

Here, we present, validate, and test a link prediction algorithm that does not require information on network structure for training, extending the problem of link prediction in social networks to bipartite networks. This is important, as network structure is often dynamic, and generalizing link prediction to novel or changing networks is necessary for some applications (e.g., forecasting the most probable prey items or parasites of a novel host species to the network). Our approach allows for the ranking of node characteristics, which can enhance our understanding of what determines the likelihood of species interactions, and for the prediction of cryptic interactions, which can influence network structure.

In our small mammal-parasite network, we determined that host litter size, parasite genus, and host diet breadth were the top three most important predictors of host-parasite interactions. Host litter size was the most important interaction predictor, suggesting the importance of host life history traits. Because host litter size is linked to other aspects of host biology known to alter parasite burdens, such as host metabolic rate [52], we suspect that the importance of litter size in this analysis may reflect an aspect of the host species’ pace of life [53, 54]. The second most important variable to our predictive model was parasite type (i.e., arthropod, helminth, or protozoa), which accounts for unmeasured differences among parasite species in their transmission or host preferences. Lastly, host habitat breadth, which can influence contact rates with parasites was an important variable in our model. Interestingly, despite the previously documented importance of host phylogenetic distance in predicting parasite community similarity [10], we found no evidence that host phylogeny improved predictive accuracy in this system. The inclusion of some covariates actively detracted from model performance, a phenomenon not observed in simulated data. This is likely a result of the low information content of these variables, or could signal the influence of variable interactions on model predictive accuracy.

Our algorithm predicted that between 110 and 157 links were missing from the network. When these links were added based on their suitability score, several network properties changed, including nestedness, togetherness, variance-to-mean ratio, and checkerboard score. While the ability to detect nestedness fluctuated with link addition, the other three metrics of network interaction patterns demonstrated consistent declining trends. This suggests that the interaction patterns became less clumped (as indicated by the checkerboard score), parasite communities became less dissimilar (as indicated by togetherness), and less aggregated (as indicated by variance-to-mean ratio). Taken together, these findings suggest link addition was not confined to species that already had many links, otherwise the variance-to-mean ratio wouldn’t have been reduced. Instead, the addition of missing links reduced overdispersion commonly observed in many host-parasite networks (including in Fig 2).

Ecologists have long recognized the issue of incomplete sampling leading to imperfect detection [55], but only recently have studies of ecological networks addressed this issue [2, 17, 56]. Here, we present an algorithm capable of accurately reconstructing a network using information on interactor traits, and predicting interaction likelihoods. This overcomes the problem of imperfect detection, and allows for the forecasting of the most probable links in ecological networks. Other approaches for the link prediction problem in bipartite networks exist. For instance, recent Bayesian approaches have used occupancy models [17] and Dirichlet network distributions [57]. However, these approaches are largely used to address slightly different problems. The first is an attempt to combine occupancy models with metacommunity analysis, predicting missing links as a means to correct error, and not for the sake of predicting unknown links. The second was developed to predict links in integer-based directed networks, and was developed under the assumption that nodes have repeated and directed interactions, such as a network of email correspondence among a group of people. Extensions of this approach could potentially support binary bipartite networks as we have examined. Another approach, the matching-centrality method [29], allows for the accurate forecasting of unobserved links in both unipartite and bipartite networks. Our approach differs in that we rely solely on trait matching between bipartite interactors to predict interaction probability, meaning that the algorithm is insensitive to network structure (allowing for increased flexibility). Lastly, by relying on host and parasite traits, our approach may provide insight into what host traits, parasite traits, or trait combinations promote the likelihood of a host-parasite interaction, and further provides a way to quantify the relative importance of host and parasite traits to interaction patterns.

Extensions of our current approach could disentangle the effect of disproportionate sampling effort, as well as other host and parasite traits, to provide a more complete understanding of what controls host-parasite interactions. This trait-based approach can be applied to other bipartite networks (e.g., plant-pollinator), as well as to spatial networks (e.g., metapopulations). The incorporation of missing links into networks that change seasonally or are logistically difficult to sample provides a more accurate description of network interactions. Further, the incorporation of these interactions may change basic network properties in non-random ways. The functional consequences for revising our understanding of ecological networks are not currently known.

## Supporting information

S1 Methods Supplemental text describing model performance on simulated data. (PDF)

S1 Fig Predictive accuracy of the plug-and-play algorithm on 1000 simulated networks, trained on 5 host and parasite traits, with an average connectance of 0.2. (PDF)

S2 Fig Predictive accuracy of the plug-and-play algorithm was not strongly influenced by the fraction of the network data that was unobserved. Specifically, these included presence and absence points, and were not included during any part of model training. This suggests that only 50% of the network can be censused, and our approach still manages to reconstruct the network with high accuracy. For these simulations, we used 5 host and parasite traits, and a connectance of 0.2).
(PDF)

S3 Fig The influence of matrix size on predictive accuracy of trained models. The color gradient corresponds to AUC values, and the axes to the number of hosts and parasites in the network.
(PDF)

S4 Fig The influence of binary trait variables on predictive model performance. Models were trained with 20 host and parasite variables on 1000 simulated networks for each fraction of binary trait value treatment. Model performance was reduced as a function of converting continuous traits to binary, but models trained on completely binary data still had high predictive accuracy.
(PDF)

S5 Fig The influence of the number of traits used to train models on predictive accuracy. At low trait numbers, predictive accuracy is reduced, but this effect is reduced after three host and parasite traits are examined.
(PDF)

S6 Fig The influence of network connectance on predictive accuracy. Low connectance increases the variability in predictive accuracy, but not the mean accuracy.
(PDF)

S7 Fig Random uninformative variables can sometimes affect model performance. Our trained models were insensitive to the addition of uninformative variables, as we added up to 50 random variables without any influence on model performance.
(PDF)

S8 Fig Models were trained using randomized trait variables, such that variables should be uninformative, and model performance should converge to an AUC of 0.5. Model performance stayed around 0.5 when models were trained on a range of random trait variables.
(PDF)
