# Supplementary material for: Predicting cryptic links in host-parasite networks
Source: PLoS Comput Biol. 2017 May 25;13(5):e1005557. doi: 10.1371/journal.pcbi.1005557 (PMC5466334; doi:10.1371/journal.pcbi.1005557)
Supplement: S1 Methods — (PDF) [file pcbi.1005557.s001.pdf]

Supplementary Material: Predicting Cryptic Links in Host-Parasite Networks

Tad Dallas[1,2,*], Andrew Park[1,3], John M Drake[1,3]

1 University of Georgia, Odum School of Ecology, Athens GA, USA
2 University of California, Environmental Science and Policy, Davis, CA, USA
3 University of Georgia, Center for the Ecology of Infectious Diseases, Athens GA, USA

*** tdallas@ucdavis.edu**

Model performance on simulated data

We performed a series of tests on our modeling approach in order to validate the `plug and play` algorithm, and to determine when estimation of missing host-parasite interactions may not be detectable. For the following, simulations were run 1000 times for each covariate level for 30 host species and 20 parasite species, and a connectance value of 0.2 unless otherwise noted.

On a series of 1000 simulated networks detailing the interactions of 30 host species and 20 parasite species, the `plug and play` method performed well, with an average AUC value of 0.95 (Fig S1). This level of accuracy was largely unaffected by training the model on less data (Fig S2), suggesting that this method can be applied regardless of sampling effort. The combination of the number of host and parasite species also did not strongly influence accuracy (Fig S3). Here, each combination of host and parasite species number (range of 10 - 30) was simulated for 100 host-parasite networks (connectance = 0.2), and we plotted the mean AUC per combination in Fig S3. Continuous trait values are not always easy to quantify, and the number of binary variables could influence model accuracy. We transformed continuous covariates into binary by considering all values of a continuous trait greater than the mean to be 1, and less than the mean to be 0. We simulated networks using 20 host and parasite traits each, and systematically changed the fraction of binary covariates out of these

20, finding that accuracy was slightly reduced when binary traits dominated (Fig S4) suggesting the importance of continuous predictors for accurate host-parasite association prediction. However, we found that as few as 3 or 4 host and parasite traits were needed in order to achieve accuracy of 0.9 (Fig S5). The relative sparcity of host-parasite associations in a bipartite network may influence predictive ability. Since few positive instances are known, it becomes difficult to estimate which interactions are most likely. However, we found connectance did not influence mean accuracy strongly, but instead influence the variance. Specifically, host-parasite networks with greater connectance had lower variance in predictive accuracy for a set of 1000 simulated networks (Fig S6). Lastly, the incorporation of random variables could influence model accuracy by reducing the detectability of important covariates for estimation of missing host-parasite associations. However, we find no evidence that the incorporation of random covariates, modeled as standard normal variates, influenced model accuracy (Fig S7). Lastly, in order to confirm that a model trained entirely on random variables would not do well in predicting missing host-parasite associations, we simulated networks based on informative trait values, but then randomized these traits (Fig S8). The result was that mean accuracy was approximately 0.5, which suggests that the model performed as well as random guessing.

For more information on model structure and to apply this approach to data, see the following figshare repository:

> Data and code to reproduce Dallas, Park, and Drake ”Predicting cryptic links in host-parasite networks” doi: 10.6084/m9.figshare.4965038

Figures

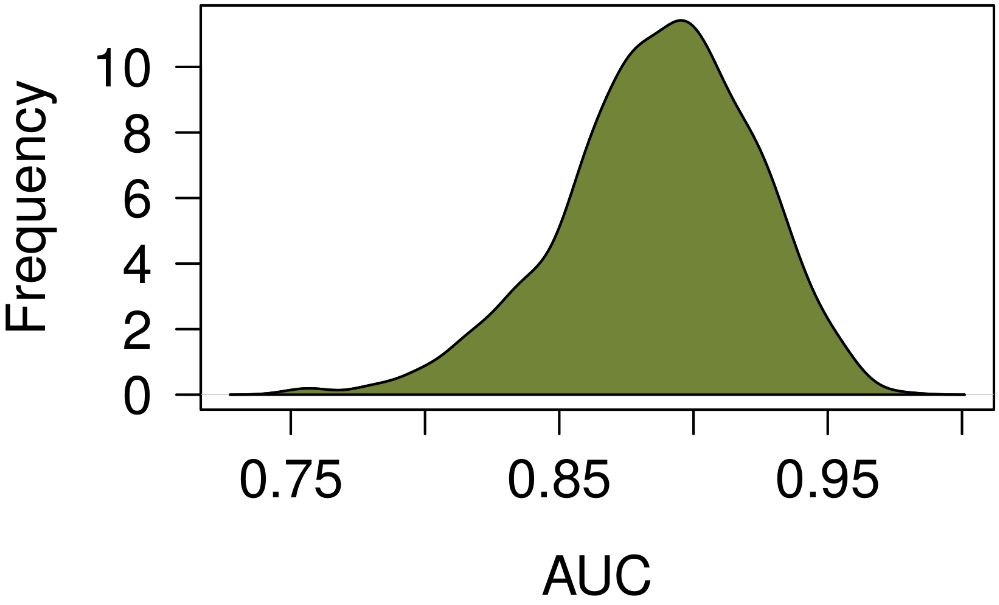

Figure S1. Predictive accuracy of the `plug and play` algorithm on 1000 simulated networks, trained on 5 host and parasite traits, with an average connectance of 0.2.

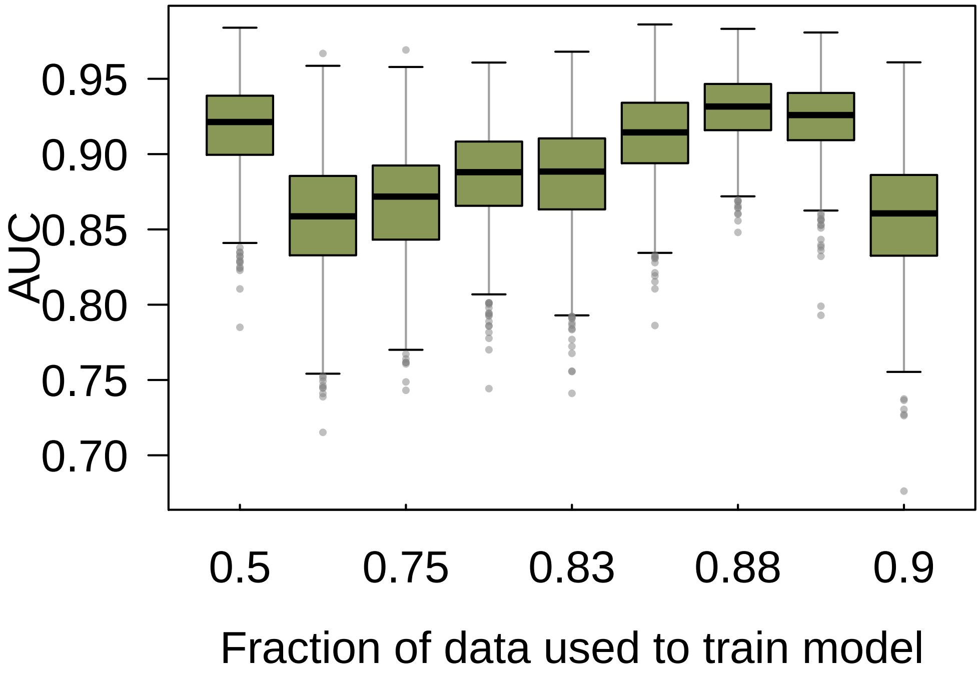

Figure S2. Predictive accuracy of the `plug and play` algorithm was not strongly influenced by the fraction of the network data that was unobserved. Specifically, these included presence and absence points, and were not included during any part of model training. This suggests that only 50% of the network can be censused, and our approach still manages to reconstruct the network with high accuracy. For these simulations, we used 5 host and parasite traits, and a connectance of 0.2).

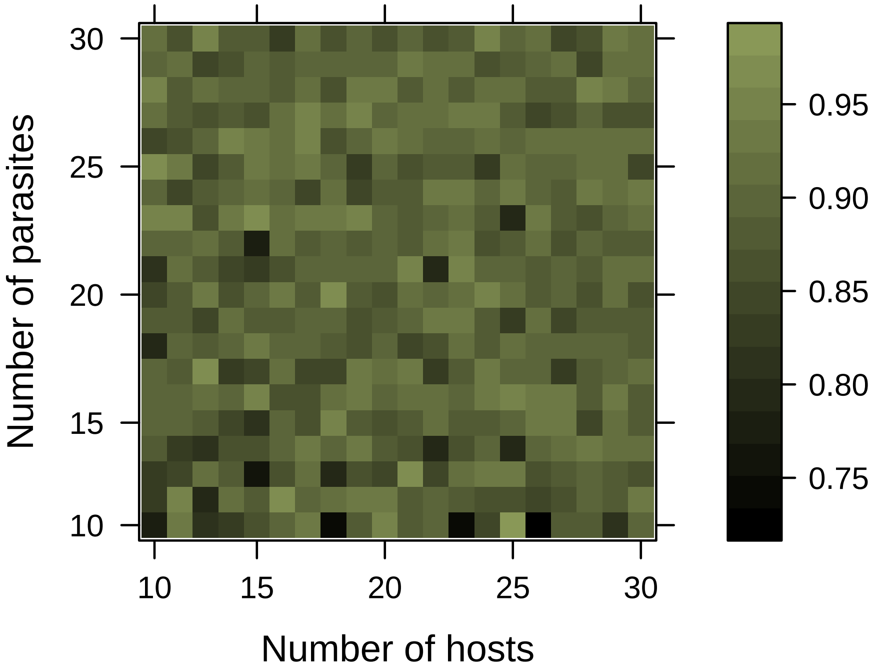

Figure S3. The influence of matrix size on predictive accuracy of trained models. The color gradient corresponds to AUC values, and the axes to the number of hosts and parasites in the network.

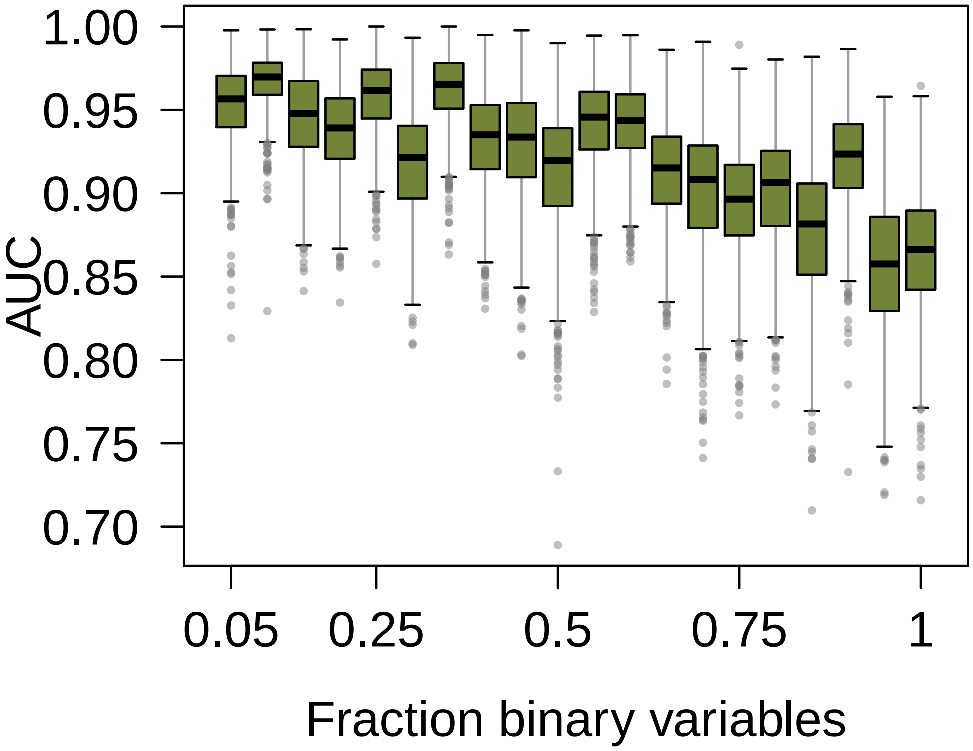

Figure S4. The influence of binary trait variables on predictive model performance. Models were trained with 20 host and parasite variables on 1000 simulated networks for each fraction of binary trait value treatment. Model performance was reduced as a function of converting continuous traits to binary, but models trained on completely binary data still had high predictive accuracy.

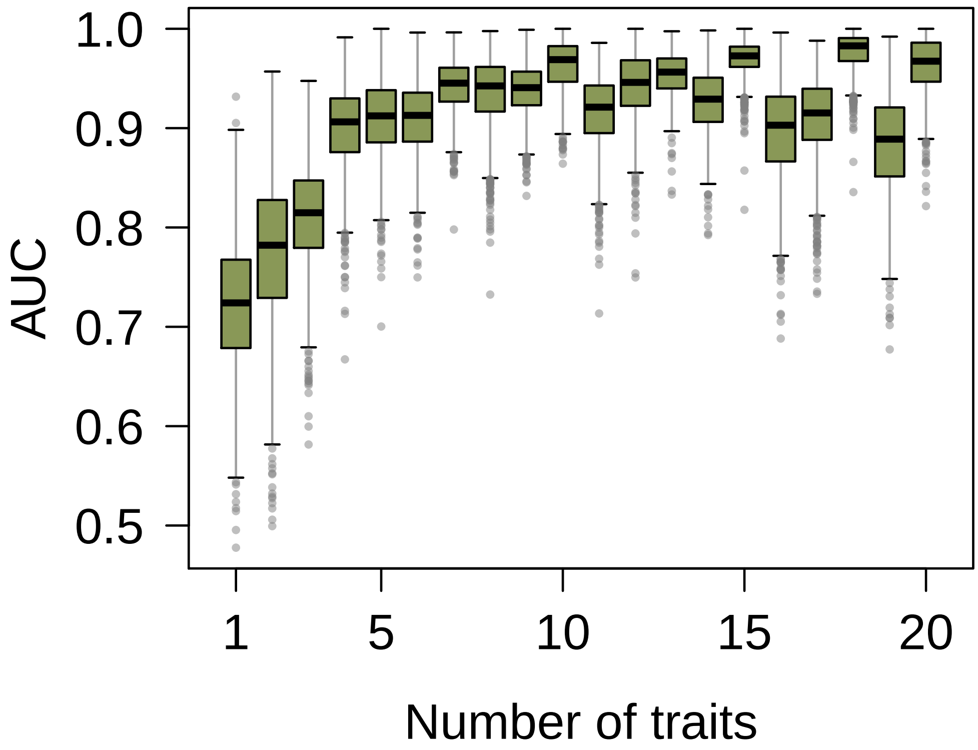

Figure S5. The influence of the number of traits used to train models on predictive accuracy. At low trait numbers, predictive accuracy is reduced, but this effect is reduced after three host and parasite traits are examined.

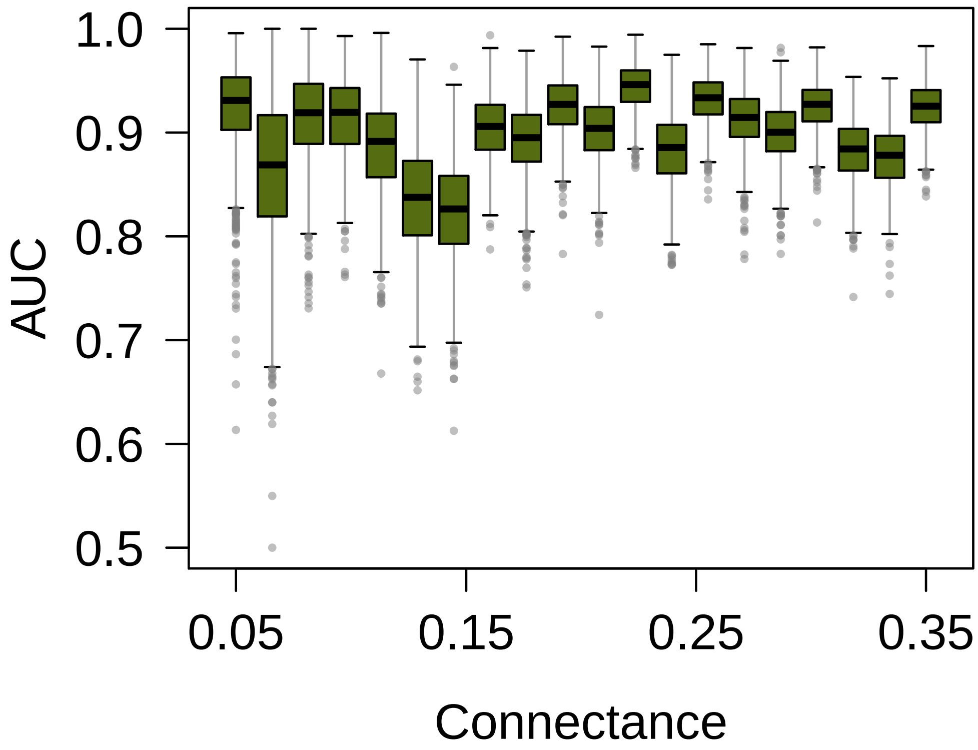

Figure S6. The influence of network connectance on predictive accuracy. Low connectance increases the variability in predictive accuracy, but not the mean accuracy.

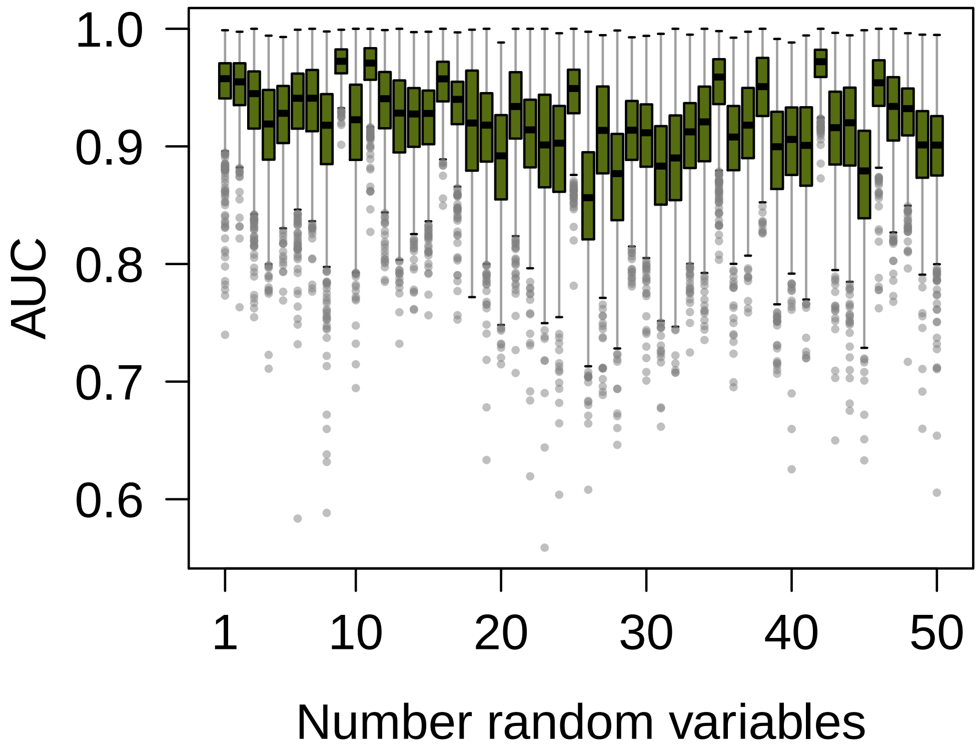

Figure S7. Random uninformative variables can sometimes affect model performance. Our trained models were insensitive to the addition of uninformative variables, as we added up to 50 random variables without any influence on model performance.

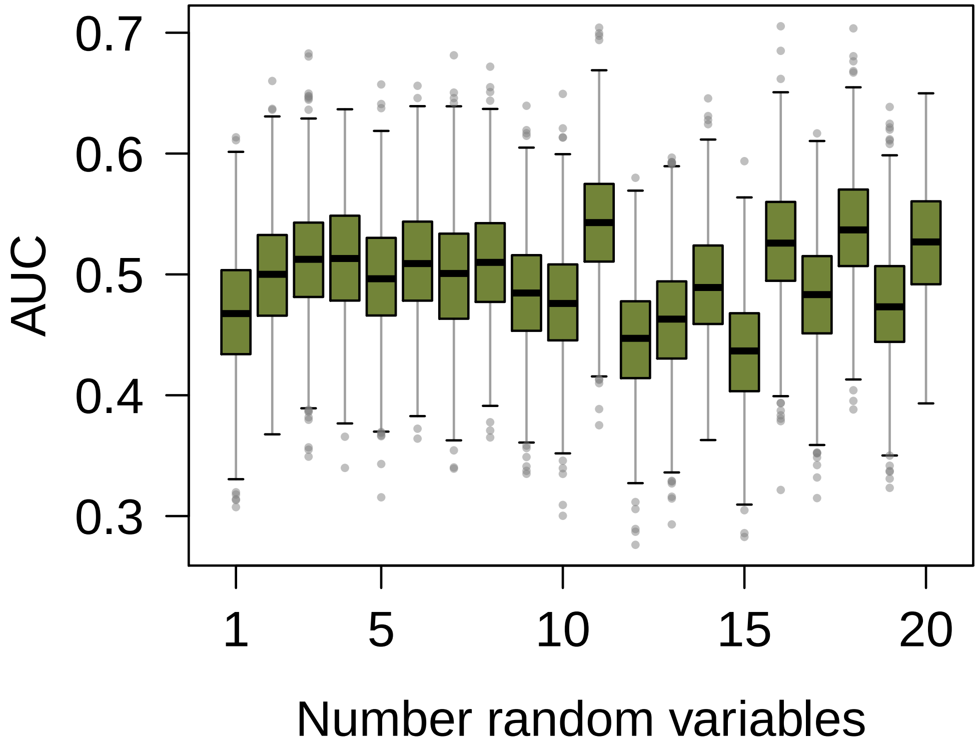

Figure S8. Models were trained using randomized trait variables, such that variables should be uninformative, and model performance should converge to an AUC of 0.5. Model performance stayed around 0.5 when models were trained on a range of random trait variables.
