# Supplementary figures and images for: Predicting cryptic links in host-parasite networks
Source: PLoS Comput Biol. 2017 May 25;13(5):e1005557. doi: 10.1371/journal.pcbi.1005557 (PMC5466334; doi:10.1371/journal.pcbi.1005557)

Frequency

10  
8  
6  
4  
2  
0

0.75

0.85

0.95

AUC

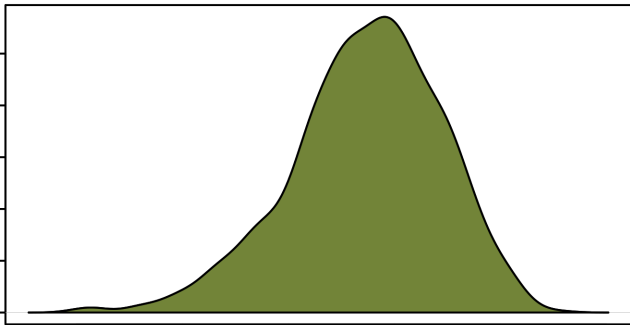

Supplement: S1 Fig — (PDF) [file pcbi.1005557.s002.pdf]

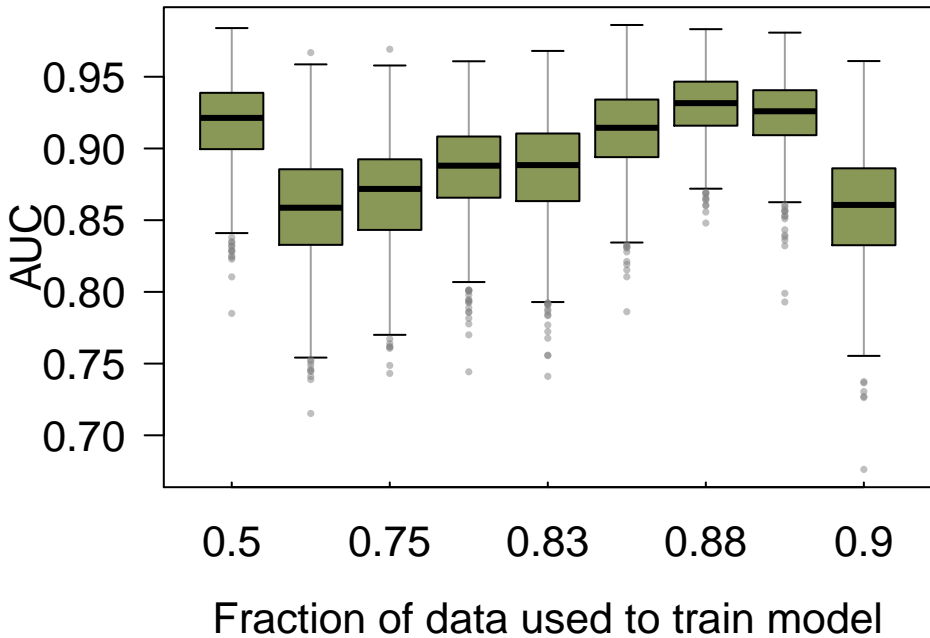

Supplement: S2 Fig — Specifically, these included presence and absence points, and were not included during any part of model training. This suggests that only 50% of the network can be censused, and our approach still manages to reconstruct the network with high accuracy. For these simulations, we used 5 host and parasite traits, and a connectance of 0.2). (PDF) [file pcbi.1005557.s003.pdf]

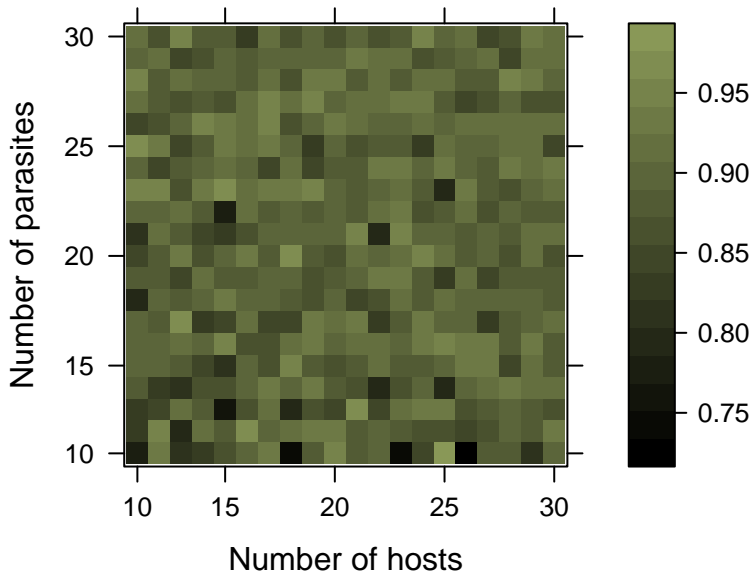

Supplement: S3 Fig — The color gradient corresponds to AUC values, and the axes to the number of hosts and parasites in the network. (PDF) [file pcbi.1005557.s004.pdf]

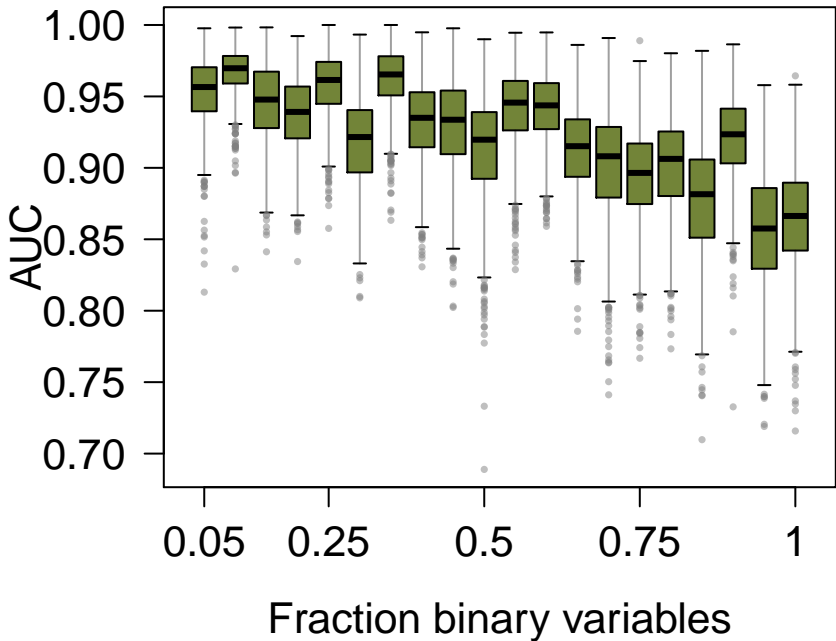

Supplement: S4 Fig — Models were trained with 20 host and parasite variables on 1000 simulated networks for each fraction of binary trait value treatment. Model performance was reduced as a function of converting continuous traits to binary, but models trained on completely binary data still had high predictive accuracy. (PDF) [file pcbi.1005557.s005.pdf]

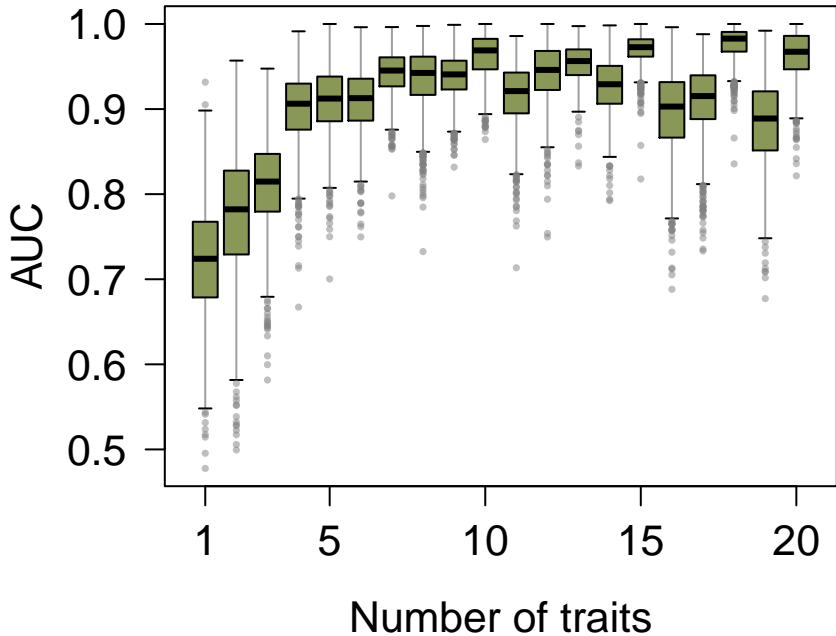

Supplement: S5 Fig — At low trait numbers, predictive accuracy is reduced, but this effect is reduced after three host and parasite traits are examined. (PDF) [file pcbi.1005557.s006.pdf]

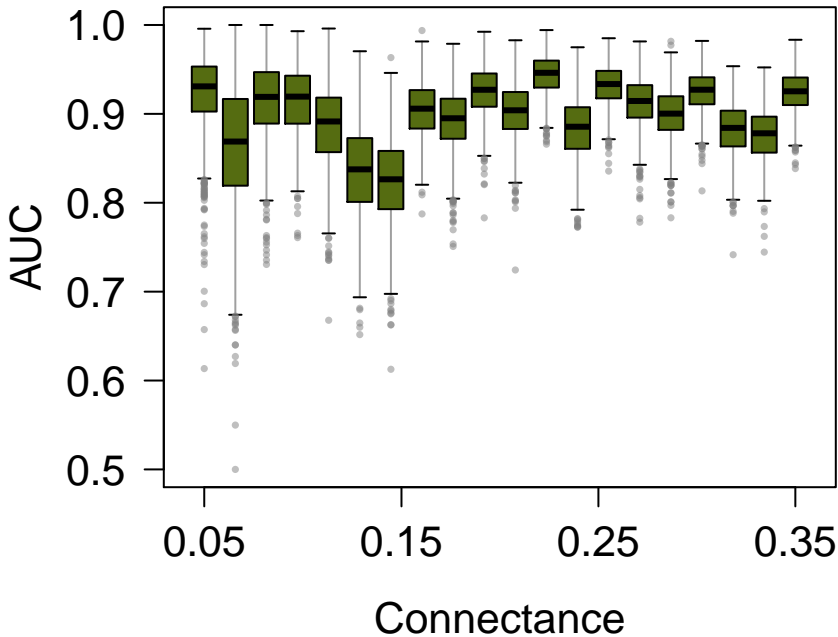

Supplement: S6 Fig — Low connectance increases the variability in predictive accuracy, but not the mean accuracy. (PDF) [file pcbi.1005557.s007.pdf]

AUC

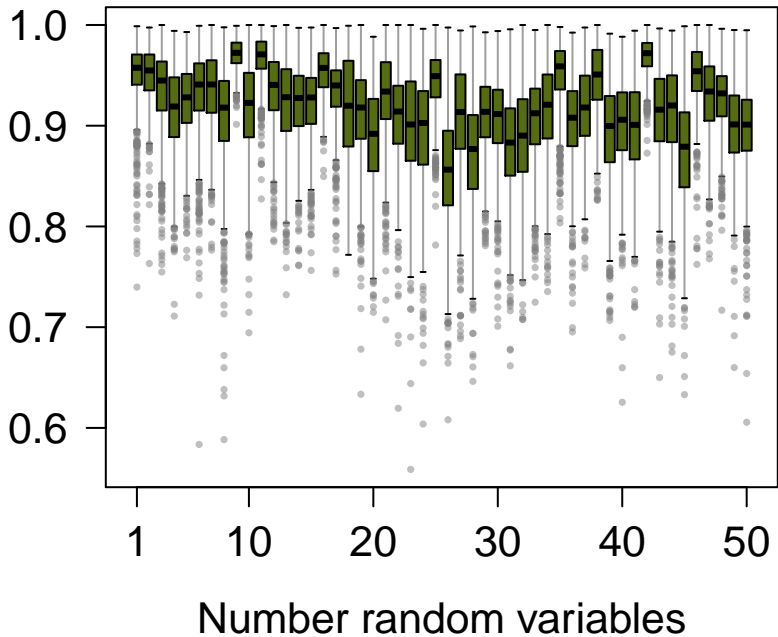

Supplement: S7 Fig — Our trained models were insensitive to the addition of uninformative variables, as we added up to 50 random variables without any influence on model performance. (PDF) [file pcbi.1005557.s008.pdf]

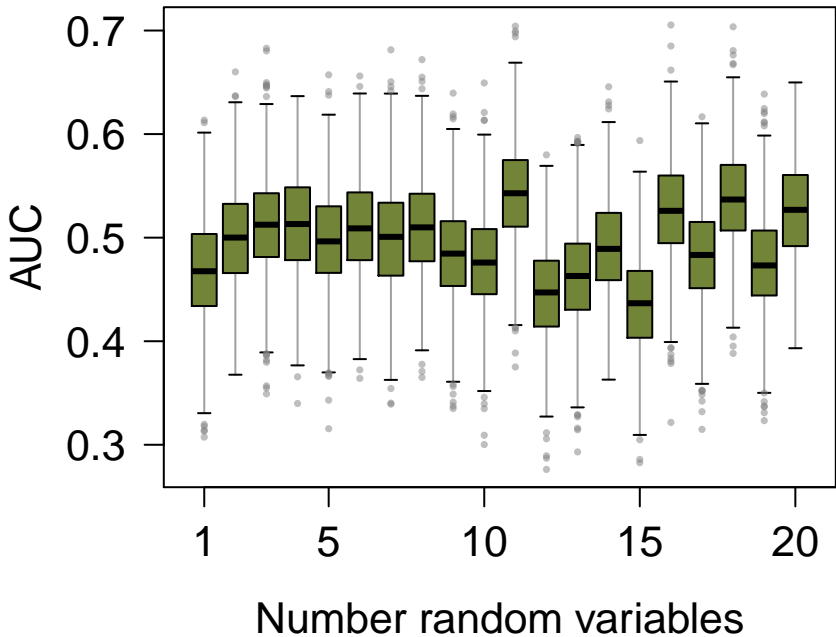

Supplement: S8 Fig — Model performance stayed around 0.5 when models were trained on a range of random trait variables. (PDF) [file pcbi.1005557.s009.pdf]
